# Bardoxolone methyl: drug development for diabetic kidney disease

**DOI:** 10.1007/s10157-020-01917-5

**Published:** 2020-06-27

**Authors:** Hironori Kanda, Kengo Yamawaki

**Affiliations:** Nephrology R&D Unit, R&D Division, Kyowa Kirin Co., Ltd. Otemachi Financial City Grand Cube, 1-9-2 Otemachi, Chiyoda-ku, Tokyo, 100-0004 Japan

**Keywords:** Bardoxolone methyl, Keap1, Nrf2, GFR

## Abstract

Bardoxolone methyl activates the Keap1/Nrf2 system that plays an important role in defense responses against oxidative stress. Importantly, bardoxolone methyl has demonstrated increases in estimated glomerular filtration rate (eGFR) in patients with diabetic kidney disease (DKD) in clinical studies. However, an overseas Phase 3 study of bardoxolone methyl in patients with stage G4 DKD was prematurely terminated due to an increased risk for heart failure, which was considered to have been caused by early-onset fluid overload. Subsequently, a Japanese Phase 2 study demonstrated, for the first time, that bardoxolone methyl directly improves GFR, which is a true indicator of kidney function, using the inulin clearance method. In Japan, bardoxolone methyl was designated for the treatment of DKD under the Priority Review and Designation (SAKIGAKE Designation) System established by the Ministry of Health, Labour and Welfare. A Japanese Phase 3 study, with endpoints such as a ≥ 30% decrease in eGFR, is currently ongoing to assess the efficacy and safety of bardoxolone methyl in more than 1,000 patients with stages G3 and G4 DKD who have no identified risk factors.

## Introduction

The prevalence of chronic kidney disease (CKD) is increasing worldwide, with primarily diabetic kidney disease (DKD), being a major public health concern. In Japan, as of 2017, approximately 335,000 patients with CKD (i.e., one out of 380 Japanese people) were undergoing chronic dialysis, making Japan notable as the country with the second highest prevalence of chronic dialysis patients in the world [1]. Diabetic nephropathy was the most frequently noted underlying disease among patients who initiated chronic dialysis in 2017, and accounted for 42.5% of these patients [1]. The medical cost of dialysis in Japan is estimated to be as high as 1.6 trillion Japanese yen (approximately US$ 17 billion) per year, which imposes a substantial burden on the national health care system.

In Japan, a limited number of drugs are approved for the treatment of CKD, including two renin-angiotensin system (RAS) inhibitors (Nu-Lotan^®^ and Tanatril^®^), which suppress urinary protein excretion, and one spherical carbon absorbent (Kremezin^®^), which lowers the level of uremic toxins. In addition, a selective vasopressin V2 receptor antagonist (Samsca^®^) was approved in Japan in 2014 as the first drug to slow the progression of autosomal dominant polycystic kidney disease (ADPKD). In many cases, the therapeutic effectiveness of these drugs is limited, and they have been reported to primarily suppress kidney function decline [2–5]. Thus, no specific drug that results in improvement of kidney function is currently available for the treatment of CKD, and there is an urgent need to develop novel drugs.

This article outlines the development of bardoxolone methyl, which shows promise as a novel therapeutic drug for CKD, including DKD.

### Bardoxolone methyl and the Keap1/Nrf2 system

Bardoxolone methyl is a semi-synthetic triterpenoid developed from the natural product oleanolic acid scaffold, and strongly activates the Kelch-like ECH-associated protein 1 (Keap1)/nuclear factor erythroid 2-related factor 2 (Nrf2) system [6]. The Keap1/Nrf2 system is activated by reactive oxygen species and electrophiles, and plays a central role in the host defense and responses against various stresses, including oxidative stress.

Dr. Yamamoto et al. have previously elucidated the exact mechanism of action of this system [7]. Keap1/Nrf2 signalling promotes transcriptional induction of antioxidative proteins, phase II xenobiotic metabolising enzymes and phase III transporters. Nrf2 is expressed, to varying degrees, in a ubiquitous manner in various organs and cells throughout the body, and has been reported to have the highest expression in the kidneys [8].

**Fig. 1:**
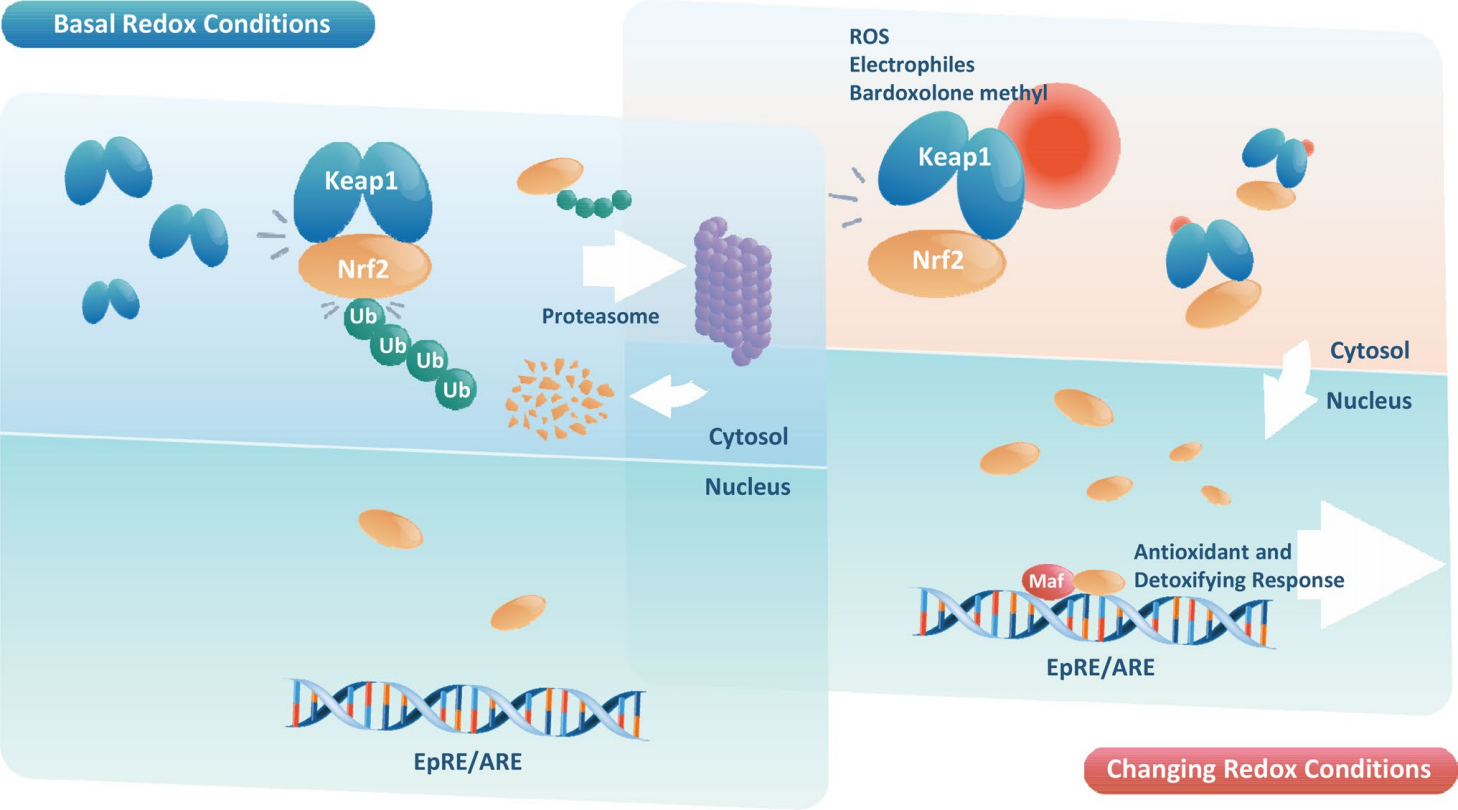
Keap1/Nrf2 system. *EpRE/ARE* electrophile responsive element/antioxidant responsive element; *ROS* reactive oxygen species; *Ub* ubiquitin

In a stress-free environment, the stress sensor protein Keap1 coordinates the ubiquitination of the transcription factor Nrf2 by Cul3-Rbx1 ubiquitin ligase. Thus, Keap1 negatively regulates Nrf2 in the cytoplasm, and the ubiquitinated Nrf2 is degraded by the proteasome, resulting in the suppression of Nrf2 function. On the other hand, when cells are exposed to bardoxolone methyl, following mechanism of action is considered based on past research [6] (Fig. 1). Bardoxolone methyl covalently binds to the reactive cysteine residue(s) of Keap1, releasing Nrf2 from the proteasome pathway into the nucleus. Subsequently, the Nrf2 translocated to the nucleus forms a heterodimer with small Maf, followed by transcriptional activation of a variety of downstream genes including antioxidant and detoxifying response through the electrophile responsive element (EpRE) or the antioxidant responsive element (ARE). In addition, it inhibits proinflammatory signalling pathways (e.g., activation of nuclear factor κB [NF-κB]) to protect the tissue against excessive oxidative stress and inflammation.

Thus, cells having the Keap1/Nrf2 system exhibit a rapid response to intracellular stress by synthesising and degrading Nrf2.

### Keap1/Nrf2 system and kidney disease

The precise mechanism of CKD progression has not yet been fully understood. It is suggested that when CKD progresses to some extent, it undergoes the final common pathway leading to end-stage kidney disease. Oxidative stress and inflammation are reported to be major factors linked to the final common pathway [9]. Reduction in glomerular filtration rate (GFR), an indicator of kidney function, is related to impaired dilation of the glomerular endothelium due to excessive oxidative stress and inflammation, mesangial cell contraction, enlargement of the mesangial area, accumulation of the extracellular matrix, and tubulointerstitial fibrosis.

Several clinical trials conducted to date in the US and Europe have been designed to explore the effects of antioxidants such as vitamin E, N-acetyl cysteine and coenzyme Q10 to treat kidney diseases [10–12]. However, these studies had a relatively small sample size for a clinical study design, and the results were contradictory. Currently available evidence does not clearly elucidate the benefits of antioxidant therapy in CKD. This may be because the potency of the currently available antioxidant therapies is not sufficient to achieve the level required for treatment.

Numerous studies using genetically modified animals (e.g., Nrf2 knockout animals, Keap1 knockdown animals) have reported the relation between Nrf2 and kidney disease [13]. Nrf2 knockout animals demonstrated the worsening of lupus-like nephritis, streptozotocin-induced kidney disease, ferric nitrilotriacetate-induced nephrotoxicity, and ischemia–reperfusion-induced kidney injury. On the contrary, Keap1 knockdown animals demonstrated improvements in kidney tubular injury due to ischemia–reperfusion injury and reduction in kidney fibrosis due to unilateral ureteric ligation. In addition, studies have shown that the activity of Nrf2 is lowered in CKD [14], and that Nrf2 could have a central role in inflammation and metabolic pathways associated with GFR in CKD [15].

Many studies have evaluated the efficacy of Nrf2 activators for the treatment of kidney injury [16]. In line with the studies using genetically modified animals, these studies also reported that Nrf2 activation leads to reduction in kidney injury, thereby suggesting the importance of this pathway in the treatment of kidney disease.

### Overseas phase 2/phase 3 studies (BEAM/BEACON studies)

In 2006, Reata Pharmaceuticals, United States, began the development of bardoxolone methyl as an anti-cancer agent. During the course of clinical studies, it was observed that bardoxolone methyl clearly increased the eGFR [17].

**Fig. 2:**
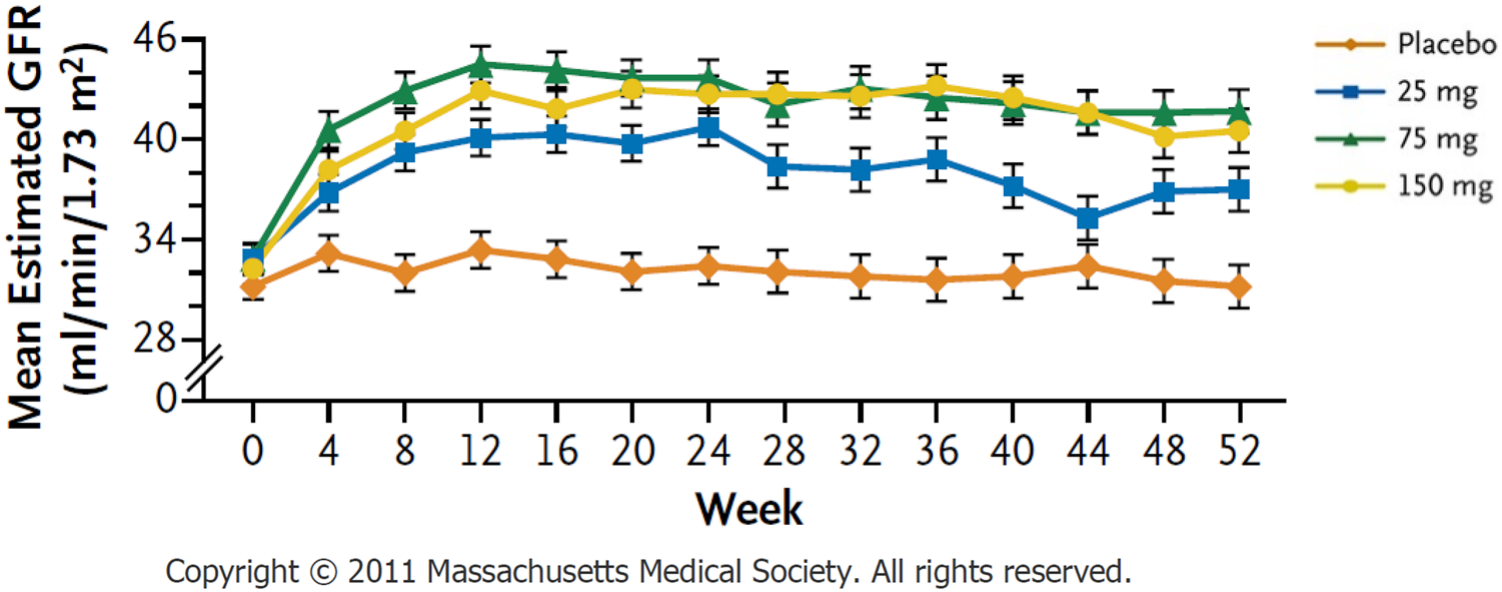
Effect of bardoxolone methyl on eGFR in the BEAM study (adapted from Pergola PE et al., with slight modification). *eGFR* estimated glomerular filtration rate

Subsequently, it was decided to investigate the possibility of a therapeutic effect of bardoxolone methyl on kidney disease. The BEAM study, a placebo-controlled, double-blind, Phase 2 clinical study in the United States, was conducted to assess the efficacy and safety of bardoxolone methyl in 227 patients with stages G3b and G4 DKD for 52 weeks [18]. In all the bardoxolone methyl dose groups (25 mg, 75 mg, and 150 mg in crystalline formulation), eGFR remarkably increased at 24 weeks [ΔeGFR (mL/min/1.73 m^2^) = 8.2 ± 1.5 (25 mg), 11.4 ± 1.5 (75 mg), 10.4 ± 1.5 (150 mg)] and this improvement persisted at 52 weeks [ΔeGFR (mL/min/1.73 m^2^) = 5.8 ± 1.8 (25 mg), 10.5 ± 1.8 (75 mg), 9.3 ± 1.9 (150 mg)] (Fig. 2). The eGFR values were notably greater than the baseline values even 4 weeks after the completion of treatment [ΔeGFR (mL/min/1.73 m^2^) = 0.7 ± 1.6 (25 mg), 2.5 ± 1.6 (75 mg), 2.3 ± 1.7 (150 mg)] [18]. Muscle spasms were the most frequent adverse event but generally mild, and hypomagnesemia, increases in alanine aminotransferase levels, and gastrointestinal effects were more common among patients receiving bardoxolone methyl. Weight reduction was also observed but independent of the magnitude of change in the eGFR. There was no increase in the rate of heart failure or other cardiovascular events.

However, the overseas, placebo-controlled, double-blind, Phase 3 BEACON study in patients with stage G4 DKD (using a 20 mg amorphous formulation of bardoxolone methyl, which corresponded to the 75 mg crystalline formulation used in the BEAM study) was prematurely terminated in 2012, just after completion of registration of 2,185 patients, because of safety concerns [19]. Subsequently, the results of the BEACON study demonstrated that bardoxolone methyl was associated with an increased risk of heart failure [19]. Because this study was terminated early, the effect of bardoxolone methyl in delaying progression to end-stage kidney disease was not sufficiently assessed in patients.

**Fig. 3:**
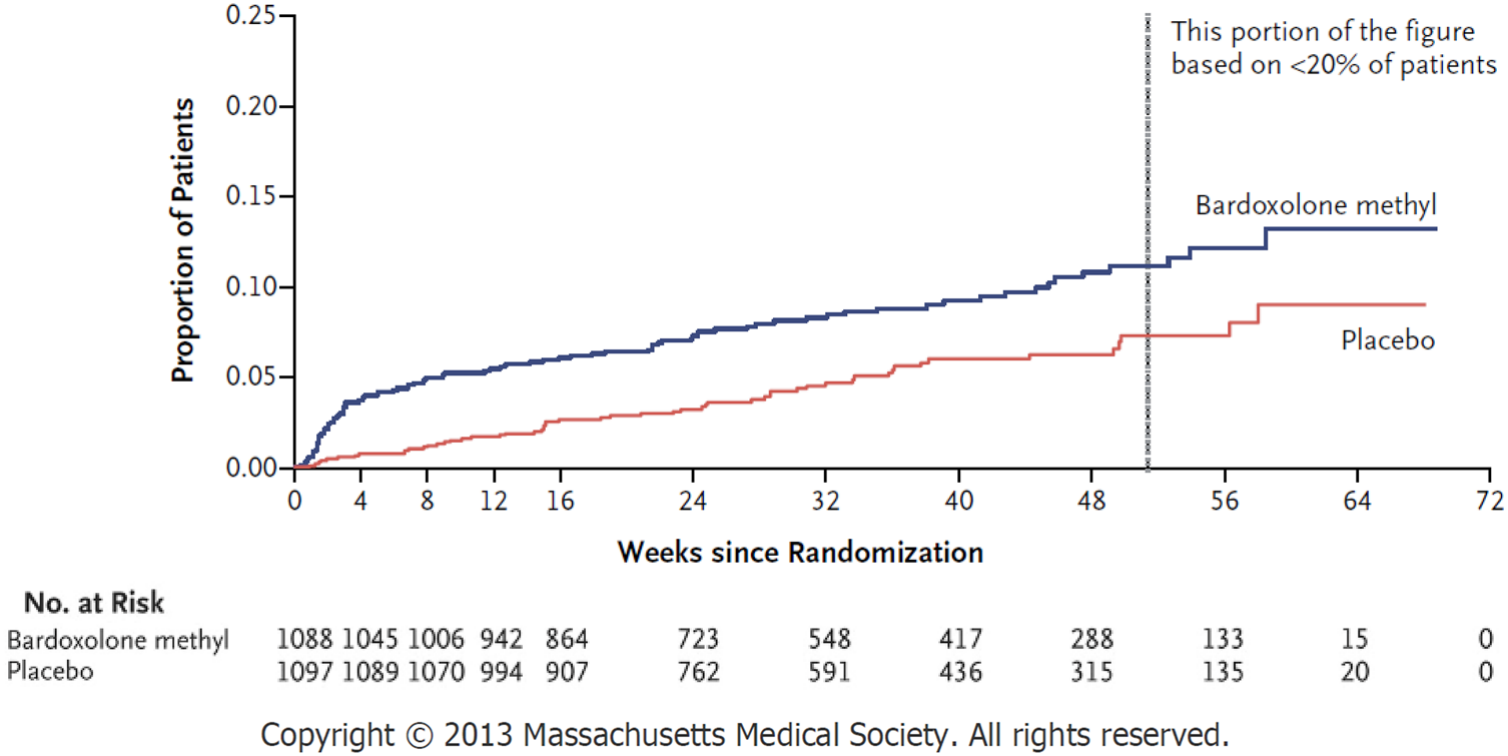
Time to occurrence of heart failure in the BEACON study (Kaplan–Meier curve) (adapted from de Zeeuw et al., with slight modification)

Post hoc analyses indicated that the events of heart failure noted in the BEACON study might have primarily been caused by fluid overload resulting from decreased sodium excretion early in the course of treatment at 4 weeks [20] (Fig. 3). In addition, risk factors were identified as high brain natriuretic peptide (BNP) levels (> 200 pg/mL) at baseline and a history of hospitalization due to heart failure [21]. The decreased sodium excretion might have been partly caused by inhibition of endothelin signalling in the kidney tubules [20]. The post hoc analyses from BEACON study also suggested that bardoxolone methyl may delay the onset of ESKD in CKD patients [22].

### Japanese phase 2 study (TSUBAKI study)

The incidences of cardiovascular events seem to be lower in CKD patients in Japan than those in the US [23]. In addition, no significant safety issue including heart failure had been observed in studies with bardoxolone methyl conducted in Japan. Considering all the factors involved, Kyowa Kirin Co., Ltd. decided to conduct a new phase 2 study (TSUBAKI study). The TSUBAKI study, initiated in 2015, was a placebo-controlled, double-blind, Phase 2 clinical study conducted to evaluate the safety and efficacy of bardoxolone methyl in Japanese patients with DKD having no risk factors for heart failure, that is, excluding high BNP levels (> 200 pg/mL) at baseline or a history of heart failure. Enrolled patients received oral bardoxolone methyl once daily for 16 weeks under careful monitoring for safety (with a titration scheme in individual patients from a starting dose of 5 mg–15 mg amorphous formulation of bardoxolone methyl). The TSUBAKI study was the first study conducted in Japan after the discontinuation of the BEACON study in patients with stage G4 DKD, and therefore, the eligibility criteria for the TSUBAKI study were limited to patients with stage G3 DKD at the beginning of the study.

Multiple clinical studies of bardoxolone methyl have demonstrated remarkable increases in eGFR and suggested a possibility that bardoxolone methyl may have an effect on creatinine metabolism to increase eGFR. For the purpose of ruling out this possibility, the primary efficacy endpoint in the TSUBAKI study was defined as the change from baseline in GFR at week 16. The GFR was measured by the inulin clearance method, which is the gold standard for GFR measurement to assess kidney function. The target sample size for the per protocol set (PPS) was 72 patients. As defined in the protocol, an interim analysis was planned to be conducted on the primary efficacy endpoint when half of the PPS was enrolled.

**Fig. 4:**
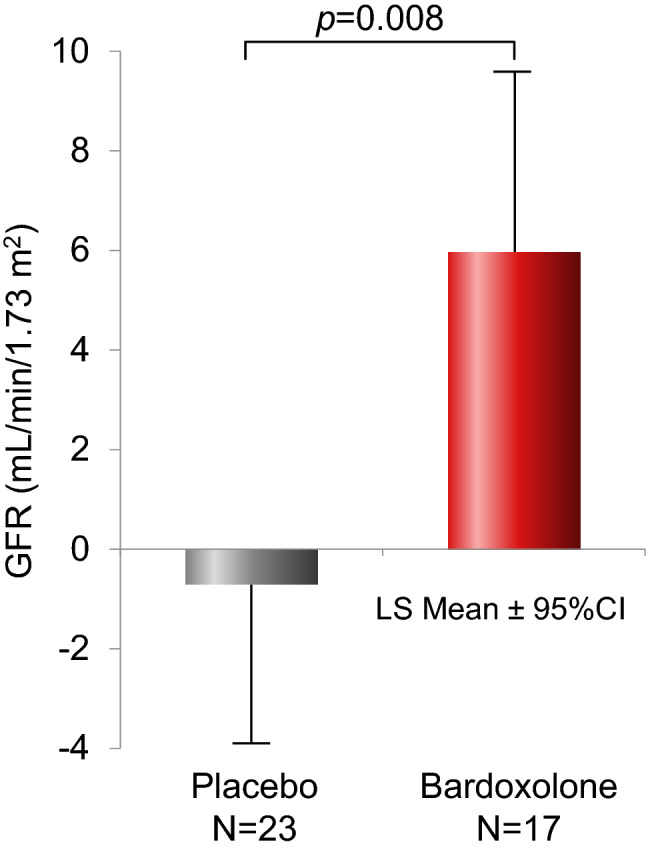
Effect of bardoxolone methyl on GFR in the TSUBAKI study (adapted from Nangaku M et al., with slight modification). LS mean was adjusted by baseline eGFR and baseline ACR. *ACR* albumin-to-creatinine ratio; Bardoxolone, bardoxolone methyl; *CI* confidence interval; *eGFR* estimated glomerular filtration rate; *GFR* glomerular filtration rate; *LS* least square

The Independent Data Monitoring Committee assessed the interim analysis results for the primary endpoint after 40 patients with stage G3 had been enrolled in the PPS. There was a significant improvement in GFR in the bardoxolone methyl group (+ 5.95 mL/min/1.73 m^2^) compared with the placebo group (-0.69 mL/min/1.73 m^2^; difference in least square means: 6.64 mL/min/1.73 m^2^; one-sided *p* value: 0.008) [24] (Fig. 4). Because GFR measurements by inulin clearance are time-consuming and burdensome, once the primary endpoint was met, enrollment was continued but no additional GFR measurements were collected. Thus, the interim analysis results demonstrated the efficacy of bardoxolone methyl, with no safety concerns, in patients with stage G3 DKD, and it was decided to newly enrol stage G4 patients in the TSUBAKI study so as to address the unmet medical needs for this patient population. As a result, no deaths or events of heart failure were reported in the safety evaluation in DKD patients with stage G3 (41 treated with bardoxolone methyl and 41 in the placebo group) and stage G4 (24 treated with bardoxolone methyl and 14 in the placebo group) [24].

As described above, the TSUBAKI study demonstrated that bardoxolone methyl improves the true kidney function (GFR) in CKD patients as measured using the inulin clearance method. In addition, the risk of heart failure may be reduced by selecting patients who are at low risk of heart failure or fluid overload, by administering bardoxolone methyl at a low starting dose with gradual escalation and by performing careful monitoring and detailed tests to detect fluid overload.

### Mechanism of GFR increase

**Fig. 5:**
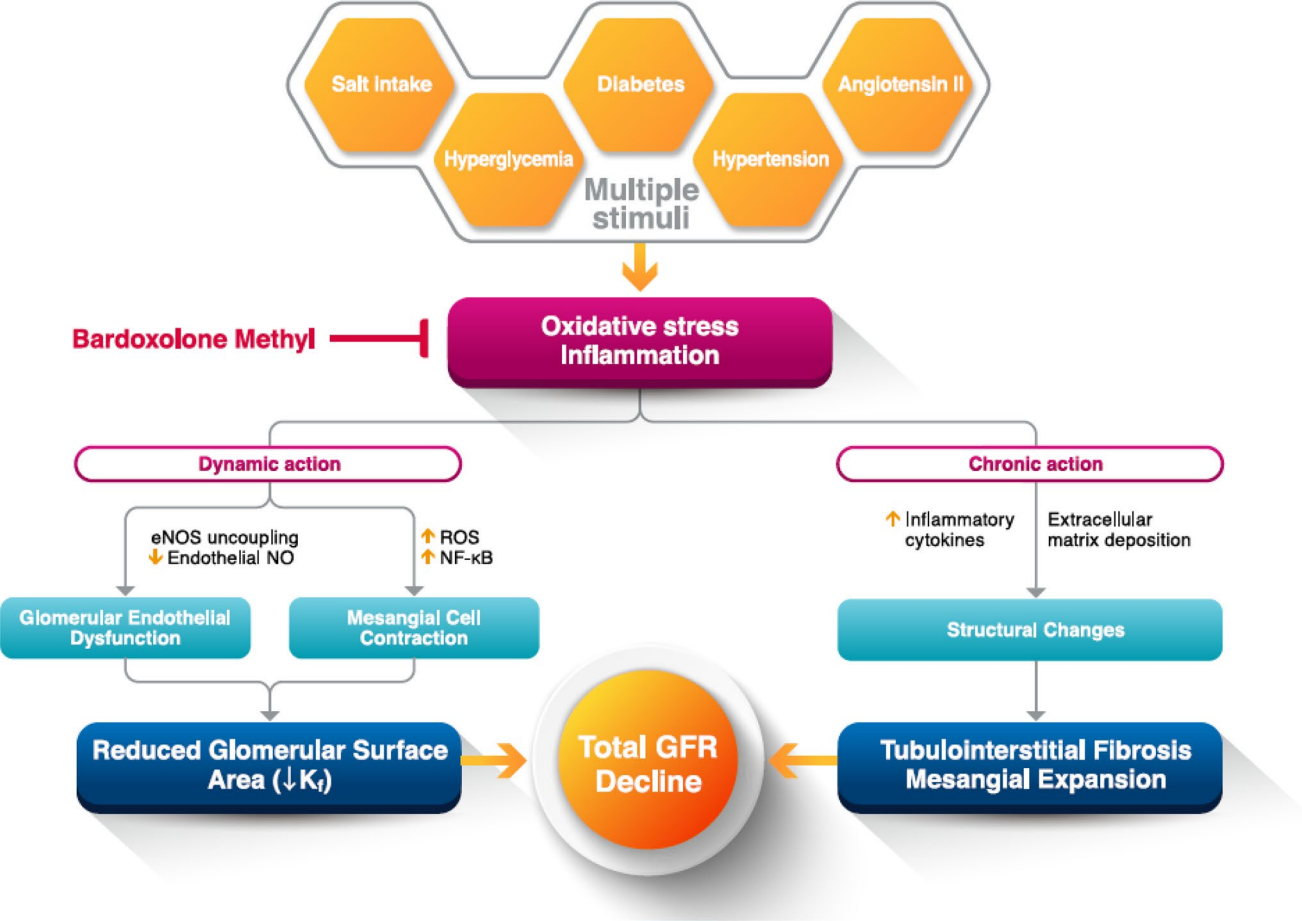
Expected mechanism for improvement of kidney function (GFR) with bardoxolone methyl (adapted from Yamawaki K et al., with slight modification). *eNOS* endothelial nitric oxide synthase; *GFR* glomerular filtration rate; *K*_*f,*_ filtration coefficient; *NF-κB* nuclear factor-κB; *NO* nitric oxide; *ROS* reactive oxygen species

The mechanism by which bardoxolone methyl improves GFR has not yet been fully understood. At present, inhibition of the inflow of calcium into mesangial cells and the resulting suppression of mesangial cell contraction [25] and improved nitric oxide (NO) bioavailability leading to maintenance of the vascular endothelial cell function [26, 27] are thought to be involved in the increased GFR observed with bardoxolone methyl (Fig. 5). An L-type calcium channel blocker has demonstrated increases in GFR, and it is considered to increase intraglomerular pressure which in turn increases GFR [28, 29]. This is a transient increase due to excessive filtration, which is clearly different from the long-lasting, sustained GFR increase over one year observed with bardoxolone methyl. The mechanism by which bardoxolone methyl increases GFR is not a transient induction of excessive filtration but may be related to different phenomena as described above. Importantly, a recent study in mice has found that Nrf2 activation increases GFR without affecting the afferent/efferent arteriole ratio [30]. In addition, when considering the fact that the effect of bardoxolone methyl in increasing GFR is sustained for a long period of time, there is a possibility, as noted in various types of animal models [31–34], that bardoxolone methyl may be effective in delaying the disease progression of structural change of the kidneys.

Bardoxolone methyl has a wide range of anti-oxidative and anti-inflammatory effects as described above and may therefore act on both dynamic changes including impaired dilation of the glomerular endothelium and mesangial cell contraction, and chronic changes including enlargement of the mesangial area, accumulation of the extracellular matrix, and tubulointerstitial fibrosis so as to improve the kidney function. Although non-clinical studies of bardoxolone methyl are challenging because the drug is known to produce toxic metabolites in rodents [35], further research is required for understanding its mechanism of action in more detail. In addition, promising results are expected from ongoing, long-term clinical studies.

### Japanese phase 3 study (AYAME study)

The AYAME study, initiated in May 2018, is a placebo-controlled, double-blind, Phase 3 clinical study assessing the efficacy and safety of bardoxolone methyl in more than 1,000 Japanese patients with stages G3 and G4 DKD without elevated BNP or a history of heart failure. Enrolled patients received once-daily oral doses of bardoxolone methyl (5 mg, 10 mg, and 15 mg amorphous formulation). On the basis of the Guideline of Clinical Endpoints in Trials of Patients with Chronic Kidney Disease [36] jointly established by the Japanese Society of Nephrology and the Japan Diabetes Society in 2018, the primary endpoint of the AYAME study is “the time to onset of a ≥ 30% decrease in eGFR from baseline or end-stage kidney disease.”

Registration of subjects in the AYAME study was completed in June 2019, and the results are expected to be released after a follow-up period of two to three years, in March 2022 at the earliest.

## Conclusion

The TSUBAKI study using the inulin clearance method demonstrated that treatment with bardoxolone methyl significantly increases measured GFR. In 2018, on the basis of its innovativeness and novelty, bardoxolone methyl was designated for the treatment of DKD under the Priority Review and Designation (SAKIGAKE Designation) System established by the Ministry of Health, Labour and Welfare.

In light of its mechanism of action, bardoxolone methyl may be effective for the treatment of CKD as a whole. Taking into account the results on efficacy obtained from the TSUBAKI study, Reata Pharmaceuticals resumed clinical studies of bardoxolone methyl for kidney disease in 2017, and is currently performing a global Phase 2/3 clinical study (CARDINAL study) in patients with Alport syndrome, which is a progressive hereditary nephritis with extremely high unmet medical needs in various countries, including Japan. The first-year results of the Phase 3 portion of CARDINAL study were announced in November 2019; bardoxolone methyl met primary and key secondary endpoints of statistically significant changes in eGFR after 48 weeks on-treatment and 4 weeks off-treatment relative to placebo [37]. In addition, bardoxolone methyl is also being investigated in a phase 3 study in ADPKD.

In Japan, more than 40,000 patients newly started dialysis during the year 2017 alone [1]. In 2018, the Investigative Committee of Measures for Kidney Disease set a target to achieve a reduction in the annual number of patients who newly start dialysis to 35,000 or lower by 2028 [38]. The development of bardoxolone methyl is expected to provide a promising new treatment for patients with DKD and various types of kidney disease.
